# Eruptive disseminated Spitz nevi – Case report^☆^^☆☆^

**DOI:** 10.1016/j.abd.2019.01.010

**Published:** 2019-12-12

**Authors:** Pablo Vargas, Rodrigo Cárdenas, Roberto Cullen, Andrés Figueroa

**Affiliations:** a Department of Dermatology, Faculty of Medicine, University of Chile, Santiago, Chile; b Clínica Alemana de Valdivia, Valdivia, Chile; c Dermatology Service, University of Chile Clinical Hospital, University of Chile, Santiago, Chile

**Keywords:** Epithelioid and spindle cell, Immunohistochemistry, Nevi and melanomas, Nevus

## Abstract

Spitz nevus is a benign melanocytic lesion, which presents in several ways: solitary, agminated, or disseminated. The disseminated variant is uncommon; it may have a rapid evolution (the eruptive form) and be difficult to manage. This report presents the case of a 24-year-old patient with multiple papules on his limbs, which had appeared four years previously. On physical examination, 120 pink and skin-colored papules were seen, which under dermoscopy were observed to be homogeneous, pink vascular lesions. Histopathologic study revealed epithelioid cells arranged in groups or singly in the dermis and dermo-epidermal junction. They were HMB-45 positive in the superficial dermis, and Ki-67 < 1%. Given these findings, a diagnosis of eruptive disseminated Spitz nevi was made.

## Introduction

Spitz nevus is a benign melanocytic lesion, which presents in a wide variety of clinical and histopathologic expressions. Its biological behavior over time tends to be uncertain. It is classified into solitary, agminated, and disseminated forms. Disseminated Spitz nevi is a rare presentation that may have a rapid (eruptive) evolution, or may develop over a prolonged period of time, several years (non-eruptive).^1^, ^2^

This report presents the case of a Chilean patient with eruptive Spitz nevi, given the unusual nature of this clinical variant and the few reports in the literature.

## Case report

**Figure 1:**
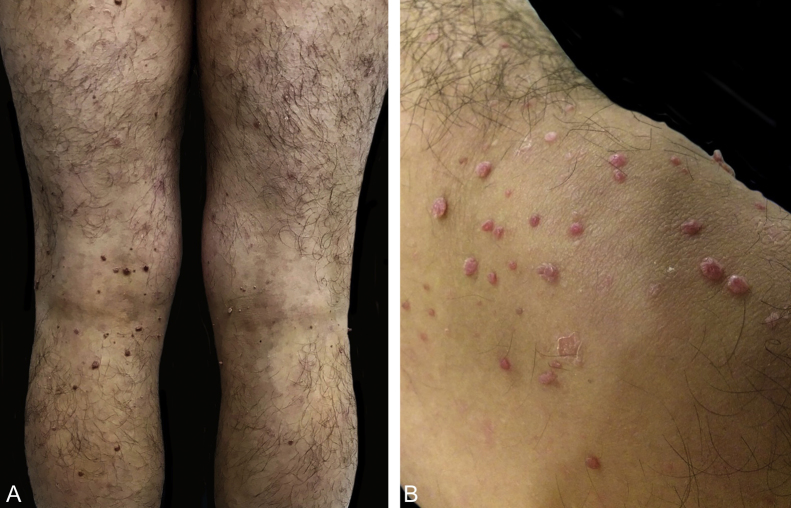
(A and B) Pink and skin-colored papules, with a warty appearance and a sessile base.

**Figure 2:**
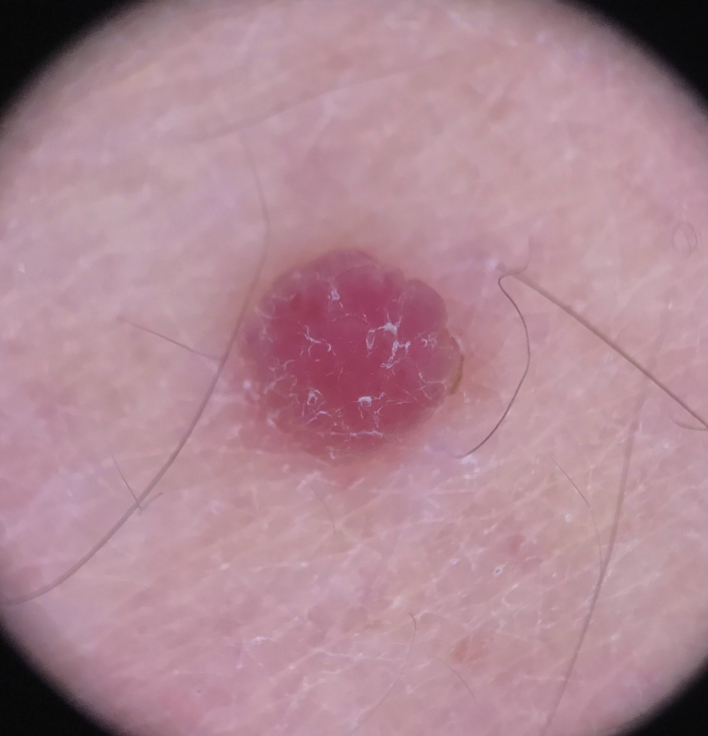
Dermoscopy: homogeneous pink vascular lesion, with small scales on the surface.

**Figure 3:**
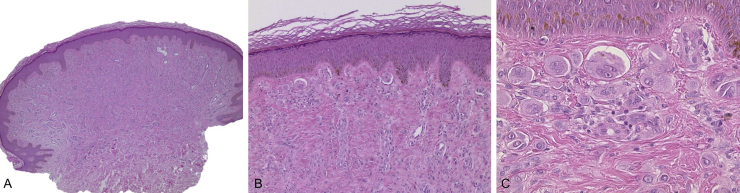
(A and B) A symmetrical and well-circumscribed compound melanocytic proliferation, arranged in epitheloid cell nests (A. Hematoxylin & eosin, ×40, B. Hematoxylin & eosin, ×100). (C) Epithelioid melanocytes with abundant eosinophilic cytoplasm, vesicular nucleus, and nucleolus (C. Hematoxylin & eosin, ×400).

**Figure 4:**
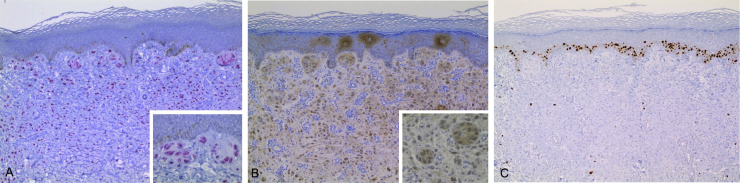
Immunohistochemical study. (A) SOX10 with a nuclear staining pattern in melanocytes. (B) S100 protein was positive. (C) S100 protein was positive.

A 24-year-old male patient, with no significant morbid history. He was referred to this hospital because of the appearance, four years previously, of multiple raised lesions on the arms, elbows, thighs, and knees. They had a rapid progressive evolution in size and number, with no mention of pain, pruritus, hemorrhage, or other problems. Some lesions, mainly on the arms, had spontaneously resolved after ten to 12 months. On physical examination approximately 120 pink and skin-colored papules were seen, with a warty appearance and a sessile base. Some were pedunculated. They were from 2 to 9 mm in diameter, and were symmetrically distributed on the knees, thighs, elbows, and arms (Fig. 1). Dermoscopy showed homogeneous pink vascular lesions, with small scales on the surface (Fig. 2). Excisional biopsy was performed on three lesions, which revealed essentially similar features: a symmetrical and well-circumscribed compound melanocytic proliferation composed of epithelioid cells with abundant eosinophilic cytoplasm arranged in groups or singly in the dermis and dermo-epidermal junction. There were variable amounts of melanin pigment, hyalinization, and vascularization. Neither significant cytological atypia nor mitosis were observed (Fig. 3). Immunohistochemical study showed SOX10 with a nuclear staining pattern in melanocytes, S100 protein was positive, and the Ki-67 labeling index was < 1% (Fig. 4). Given these findings, a diagnosis of eruptive disseminated Spitz nevi was made, and the patient agreed to the surgical excision of the main lesions under general anesthesia and strict follow-up.

## Discussion

Eruptive disseminated Spitz nevi was described in 1974 as juvenile eruptive melanoma, by Wallace et al.^3^ It is a rare pathology with only 27 reports in the literature to date.^4^ The present case correlates with those described, given that the average age for the onset of the clinical symptoms is 21 years. There are reports from newborn patients to 35-year-old adults. The majority are women and in most cases there are no trigger factors. Isolated associations have been described with chemotherapy, intravenous drugs, surgery, exposure to the sun, and pregnancy.^5^, ^6^ The lesions usually appear on the trunk and the proximal limbs. There have been four cases of spontaneous partial resolution, a condition observed in the present patient. It is of utmost importance to perform differential diagnosis, to distinguish between this and metastatic malignant melanoma, also taking into account multiple juvenile xanthogranuloma, urticaria pigmentosa, and dysplastic nevi syndrome.

In adults, a solitary Spitz nevus is usually treated by surgical removal, after clinical diagnosis, given its uncertain biological behavior. In practice, this is difficult to do in the eruptive variant, considering the number of lesions seen. Various therapeutic options have been tried, such as electrocoagulation,^7^ liquid nitrogen,^8^ and imiquimod,^9^ without success. Although malignization has not been reported, the few cases reported and its erratic evolution requires offering the patient several therapeutic options, from conservative management with clinical follow-up and regular dermoscopy, to active management with topical or surgical therapy, as is the case with the present patient.^10^

## Authors’ contribution

Pablo Vargas: Approval of the final version of the manuscript; conception and planning of the study; elaboration and writing of the manuscript; obtaining, analyzing, and interpreting the data; effective participation in research orientation; critical review of the literature; critical review of the manuscript.

Rodrigo Cárdenas: Approval of the final version of the manuscript; obtaining, analyzing, and interpreting the data; critical review of the literature; critical review of the manuscript.

Roberto Cullen: Approval of the final version of the manuscript; obtaining, analyzing, and interpreting the data; critical review of the literature.

Andrés Figueroa: Approval of the final version of the manuscript; obtaining, analyzing, and interpreting the data; intellectual participation in propaedeutic and/or therapeutic conduct of the cases studied; critical review of the manuscript.

## Financial support

None declared.

## Conflicts of interest

None declared.
